# Exploring the utility of circulating miRNAs as diagnostic biomarkers of fasciolosis

**DOI:** 10.1038/s41598-024-57704-9

**Published:** 2024-03-28

**Authors:** Sumaiya Chowdhury, Alison Ricafrente, Krystyna Cwiklinski, Dayna Sais, John P. Dalton, Nham Tran, Sheila Donnelly

**Affiliations:** 1 The School of Life Sciences, University of Technology, Sydney, Australia; 2 Centre for One Health, School of Natural Sciences, Ryan Institute, National University of Ireland Galway, Galway, Ireland; 3 School of Biomedical Engineering, Faculty of Engineering and Information Technology, University of Technology Sydney, Ultimo, NSW Australia; 4 Present Address: Institute of Infection, Veterinary and Ecological Sciences, University of Liverpool, Liverpool, UK

**Keywords:** Parasitology, Infectious-disease diagnostics, Non-coding RNAs

## Abstract

Effective management and control of parasitic infections on farms depends on their early detection. Traditional serological diagnostic methods for *Fasciola hepatica* infection in livestock are specific and sensitive, but currently the earliest detection of the parasite only occurs at approximately three weeks post-infection. At this timepoint, parasites have already entered the liver and caused the tissue damage and immunopathology that results in reduced body weight and loss in productivity. Here, we investigated whether the differential abundance of micro(mi)miRNAs in sera of *F. hepatica*-infected sheep has potential as a tool for the early diagnosis of infection. Using miRNA sequencing analysis, we discovered specific profiles of sheep miRNAs at both the pre-hepatic and hepatic infection phases in comparison to non-infected sheep. In addition, six *F. hepatica*-derived miRNAs were specifically identified in sera from infected sheep. Thus, a panel of differentially expressed miRNAs comprising four sheep (miR-3231-3p; miR133-5p; 3957-5p; 1197-3p) and two parasite miRNAs (miR-124-3p; miR-Novel-11-5p) were selected as potential biomarkers. The expression of these candidates in sera samples from longitudinal sheep infection studies collected between 7 days and 23 weeks was quantified using RT-qPCR and compared to samples from age-matched non-infected sheep. We identified oar-miR-133-5p and oar-miR-3957-5p as promising biomarkers of fasciolosis, detecting infection as early as 7 days. The differential expression of the other selected miRNAs was not sufficient to diagnose infection; however, our analysis found that the most abundant forms of fhe-miR-124-3p in sera were sequence variants (IsomiRs) of the canonical miRNA, highlighting the critical importance of primer design for accurate diagnostic RT-qPCR. Accordingly, this investigative study suggests that certain miRNAs are biomarkers of *F. hepatica* infection and validates miRNA-based diagnostics for the detection of fasciolosis in sheep.

## Introduction

The liver fluke parasite, *Fasciola hepatica*, is a trematode that causes fasciolosis, a major public health issue and neglected tropical disease affecting over 2.4 million people in over 70 countries^1–3^. Prevalence of liver fluke infection is thought to be on the rise due to the spread of drug-resistant parasites and climate change^4,5^. While *F. hepatica* has adapted to infect every mammalian host that it has encountered^2,6^, grazing ruminants are most susceptible to *F. hepatica* infection due to their ingestion of pasture and water contaminated with the infective metacercariae. As such, fasciolosis is a significant economic burden to the livestock industry, with global losses of over US $3 billion per annum^7,8^.

Within hours of ingestion, the *F. hepatica* metacercariae excyst in the gut, releasing the newly excysted juveniles (NEJ), which penetrate the intestinal wall to migrate through the peritoneal cavity to the liver. This pre-hepatic stage takes approximately two weeks, after which the pathology resulting from the parasite’s penetration into the liver becomes evident^9–11^. Fasciolosis results from the tissue damage and bleeding caused by the parasite’s burrowing and feeding in the liver and the associated immune-mediated pathology^12–14^. Within the liver, the juvenile flukes grow and mature, and by 8–12 weeks, the adult parasites enter the bile ducts to establish chronic infection, during which time they produce eggs that are excreted within the faeces, consequently contaminating pasture and continuing the life cycle^9^. Once established in their ruminant hosts, the parasites can survive for months and even years if the infection is untreated^11,15^.

Several methods are currently employed to diagnose fasciolosis in ruminants. While these techniques are effective, they have notable limitations, especially for the detection of early infection^16,17^. The gold standard for liver fluke detection is the faecal egg count (FEC), which is a preferred technique as the presence of liver fluke eggs represents a definitive diagnosis^18,19^. It is inexpensive, and both the sample collection and protocol are uncomplicated^16^. However, this method only detects infection at 8–12 weeks^20^. In addition, due to the uneven distribution of eggs in the faeces when the worm burden is low this method lacks sensitivity^21^. Coproantigen ELISAs can detect liver flukes two to three weeks earlier than FEC^22^ but this antigen-based assay lacks sensitivity due to the low and inconsistent abundance of the liver fluke antigens in the host samples^23,24^. Higher sensitivity can be obtained using serological techniques^25^. Detection of anti-liver fluke antibodies in serum is a more sensitive alternative, and ELISAs for these have been proven to detect infection from three to four weeks post-infection. However, this method cannot distinguish between new and previous infections^24^. Additionally, maternal antibodies can persist in lambs for up to 12 weeks post-birth, limiting the applicability of this assay during the first 2 months on pasture^26^. Another complication of antibody detection is the lack of specificity due to cross-reaction with antibodies raised in response to other infections^27^. Finally, although the presence of liver enzymes within sera samples, including glutamate dehydrogenase (GLDH), gamma-glutamyl transferase (GGT) and albumin, are a good indication of liver damage, these changes are transient, variable, and not specific for the liver fluke^28^.

As there is currently no effective vaccine against *F. hepatica*^29^, liver fluke control relies heavily on the available chemical drugs, such as triclabendazole (TCBZ), closantel, and clorsulon^30^. Due to the challenge of detecting pre-patent infection, farmers opt for preventative treatment measures involving blanket treatment of animals before release onto pasture and further treatment depending on factors such as housing time, local climate, fasciolosis prevalence, age of herd, and grazing period^17,31,32^. Apart from the financial burden of drenching an entire herd with flukicides, this practice is driving the emergence of drug resistance against these available chemical treatments. As such, ‘test before you treat’ strategies are now being advocated for sustainable parasite control^33–37^. If early diagnosis was possible, anthelmintics could be administered to infected animals only, which would prevent/minimise the parasites from breaching the liver capsule. This approach would reduce liver damage and avert the loss of animals. Additionally, it would decrease the number of eggs dispersed onto pasture and curtail the unwarranted use of flukicides. Thus, there is a clear industry requirement for new diagnostic tools that can reliably identify *F. hepatica* infections, particularly at the pre-hepatic phase of infection.

Micro(mi)RNAs are small non-coding RNAs that play a central role in all biological processes^38^ and offer great promise as markers of disease. Importantly, changes in their expression profiles have been correlated to several pathologies, including infectious diseases^39^. In addition, circulating miRNAs are present and highly stable in most bodily fluids, including blood, saliva, urine, and milk, making them robust candidates for non-invasive diagnostic and prognostic biomarkers^40^.

Several studies have reported alterations in the abundance of both host and parasite-derived miRNAs in circulation during helminth infections, suggesting their utility as biomarkers for detecting infection^41–44^. While the diagnostic potential of circulating miRNAs during fasciolosis has not yet been explored, we have previously shown that *F. hepatica* miRNAs are expressed in a life cycle stage-specific manner and that fluke-derived miRNAs can be detected in host cells during the pre-hepatic stage of infection^45,46^. Therefore, in this study, we explored whether a differential abundance of host and *F. hepatica* miRNAs in sera was evident in experimental infected sheep that could be used to diagnose *F. hepatica* infection, particularly from the early pre-hepatic stage of infection. This would enable prompt and effective intervention before the parasite causes significant liver pathology.

## Materials and methods

### Sheep sera samples from experimental *F. hepatica* infections

The sheep sera samples were taken from previous experimental infection studies, which were all carried out according to standardised protocols to ensure consistency and reproducibility between studies. Male sheep were orally infected with *F. hepatica* metacercariae*,* and blood samples collected by jugular venepuncture at various time points (Table 1), using appropriate blood collection tubes for serum collection. The blood was allowed to clot at room temperature, followed by centrifugation at 1000 × g for 10 min in a refrigerated centrifuge. The serum was collected and aliquoted into 1 ml aliquots that were frozen at − 80 °C. Four sets of sheep sera samples were used in this study, with each set representing an independent infection cohort (Table 1). The samples from sets A, B and D, were processed in the Molecular Parasitology Laboratory, Ireland. The samples collected in Spain (Set C) were transported to the Molecular Parasitology Laboratory in Ireland frozen on dry ice. Upon delivery, they were placed at − 80 °C. Samples used for this study were defrosted in the fridge the evening before RNA extraction; this process was the first time they had been thawed since collection. Samples from Set A and Set B were used to prepare the small RNA sequencing library. Set C (pre-hepatic time points) and Set D (hepatic time points) were used for RT-qPCR validation of the diagnostic potential of selected miRNAs. Sets C and D were obtained from longitudinal sheep *F. hepatica* infection studies that included non-infected age-matched animals as field controls.

**Table 1:** Details of the experimental *F. hepatica* infections in sheep.

Samples for sequencing		
	Set A	Set B
Study type	Cross-sectional	Longitudinal
Cohort (infection type)	Pre-hepatic infection	Hepatic infection
Sheep breed	Dorset cross sheep	Dorset cross sheep
Sheep age (at day 0)	6 months	6 months
*F. hepatica* isolate	Italian isolate (Ridgeway Research, UK)	South Gloucester isolate (Ridgeway Research, UK)
Infection dose	150 metacercariae	150 metacercariae
Collection time points	0, 2, 9, 14, 18 dpi (n = 6/timepoint)	3, 7, 10, 14 wpi, Neg-14 wpi (n = 6/timepoint)
Study location	AFBI, UK	AFBI, UK
Study reference	^47^	^47^

Samples for qPCR validation		
	Set C	Set D
Study type	Longitudinal	Longitudinal
Cohort (infection type)	Pre-hepatic infection	Hepatic infection
Sheep breed	Merino-breed sheep	Dorset cross sheep
Sheep age (at day 0)	8 months	6 months
*F. hepatica* isolate	Italian isolate	Italian isolate
Infection dose	150 metacercariae	120 metacercariae
Collection time points	0dpi (n = 3 non-infected; 9 infected) 7, 14 dpi (n = 14 non-infected; 11 infected)	3, 15, 20, 23 wpi (n = 6 non-infected; 4–5 infected/timepoint)
Study location	Cordoba, Spain	AFBI, UK
Study reference	Not published	^47^

*dpi* Days post-infection, *wpi* Weeks post-infection, *Neg-14wpi* Non-infected age-matched control at 14 weeks.

### Ethics statement

Experimental procedures at Agri-Food and Biosciences Institute (AFBI; UK) were carried out under license from the Department of Health, Social Services and Public by the Animal (Scientific Procedures) Act 1986 (License No. PPL 2771; PPL 2801), after ethical review by the AFBI Animal Ethics Committee. Experimental procedures at the University of Cordoba, Spain were carried out under license from 22-12-2015-381 in accordance with EU Directive 2012/707/UE and RD 53/2013 following ethical approval from the University of Cordoba Bioethics Committee (code no, 2015-PI-038).

### RNA isolation from sheep sera, lamb tissue, and newly excysted juveniles

Small RNA extraction was performed on sheep sera using Tri-Reagent as described previously^48^. Frozen serum samples were thawed and 400 µl aliquots were treated with proteinase K (1 mg/mL) for 20 min at 37 °C to degrade proteins. Then, 750 μL Tri-Reagent RT LS (Molecular Research, US) and 100 μL 4-bromoanisole was added to each 400 μL of serum samples to solubilise the nucleic acids. The homogenate was gently mixed and centrifuged at 12,000 × *g* for 20 min. The nucleic acids were precipitated with 5 μL of glycogen (5 mg/μL) and 500 μL of 100% isopropanol at − 20 °C overnight. The RNA precipitate was pelleted by centrifugation at 12,000 × *g*, for 15 min, washed twice with 70% ethanol and reprecipitated by centrifugation at 12,000 × *g* for 10 min, and finally re-suspended in RNase free H_2_O. For each serum sample, the RNA extracted from a total of 800 μL starting volume of sera was combined for subsequent analysis. All samples were processed at the same time, and each 400 µl aliquot was processed in the same manner. This process on average yielded 50 ng/μl of RNA per individual serum sample. The quality and quantity of RNA was assessed by POLARstar^®^ Omega Multimode Microplate Reader (for small RNA sequencing library preparation) or NanoDrop reader (for RT-qPCR).

RNA was extracted from a sample of lamb rump using RNAzol RT (Molecular Research Centre Inc, USA). Approximately 100 mg of tissue was snap-frozen with liquid nitrogen and homogenised in 1 mL of RNAzol RT with a mortar and pestle for RNA extraction, which was performed as described previously^46^ and yielded 2 μg of RNA.

The NEJ RNA had been previously isolated, as described by Ricafrente et al.^45^. NEJs were excysted using our standardised protocols. The *Fasciola hepatica* metacercariae (Italian isolate), sourced from Ridgeway Research Ltd (UK) were used for excystment and 24 h culture of NEJ. Specifically, metacercariae were removed from the visking tubing and incubated for a maximum of 10 min in 2% sodium hypochlorite with agitation at room temperature to remove the outer cyst wall. The parasites were then washed in distilled water by sedimentation to remove all traces of sodium hypochlorite. The washed parasites were re-suspended in excystment medium (1.2% sodium bicarbonate, 0.9% sodium chloride, 0.2% sodium tauroglycocholate, 0.07% concentrated hydrochloric acid, 0.006% L-cysteine) and incubated for up to 3 h at 37 °C in 5% CO_2_. NEJ were recovered using a pipette and washed several times in PBS by sedimentation to remove all traces of the excystment media. The NEJ were then transferred to pre-warmed (37 °C) culture medium (RPMI 1640 medium (ThermoFisher Scientific) containing 2 mM L-glutamine, 30 mM HEPES, 0.1% (w/v) glucose, and 2.5 µg/ml gentamycin) and incubated for 24 h at 37 °C in 5% CO2. Following the incubation, the NEJ were centrifuged at 400 × g for 5 min to pellet the NEJ and the media was removed. The NEJ pellet was washed three times with PBS and stored at − 80 °C prior to RNA extraction. This process was carried out in triplicate using 1000 NEJ per replicate. No serum was used in the culture media as these samples were also used for proteomic analyses as described by Cwiklinski et al.^49^. RNA was extracted using the miRNeasy mini kit (Qiagen) according to the manufacturer’s instructions. The QIAzol Lysis Reagent was added directly to the frozen NEJ pellet for lysis and homogenization, and the RNA eluted into a final volume of 50 µl RNase-free water. RNA integrity and concentration were verified using the 260/280 LVis plate functionality of the PolarStar Omega Spectrophotometer (BMG LabTech) and the Quant-iT RiboGreen RNA Assay Kit (TermoFisher Scientifc). The RNA was transported to University of Technology, Sydney frozen on dry ice and stored at − 80 °C upon delivery.

### Sequencing and bioinformatics

RNA extracted from each time point (representing six animals) was pooled into a single representative sample to provide sufficient yield of RNA for sequencing. RNA library preparation was performed by Macrogen Oceania (NSW, Australia) using 1 μg of total RNA with the TruSeq Small RNA library Prep Kit according to manufacturer’s instruction (Part#15,004,197 Rev. G) and sequenced using Illumina NextSeq 500. The quality of the raw FASTq files was assessed using FastQC^50^, and consequently the adaptor sequences were excised, and low-quality sequences were removed (< 20 phred score, and short read length < 18 nt) using the bioinformatic tool CutAdapt (v3.4)^51^. The cleaned sheep sera sequence reads were then aligned against known sheep mature miRNAs from miRBase: *Ovis aries* (Oar_V4.0) using Bowtie (v1)^52^, allowing zero mismatches. The miRNA sequence reads that did not align against the sheep miRNAs were then aligned against *F. hepatica* mature miRNA sequences from miRBase (Fhepatica_v1) and other published sources^45,53–55^ (Supplemental Table 1) allowing zero mismatches. The counts for the sheep and *F. hepatica* miRNAs were extracted from the resultant Sam files using SamTools^56^.

### Characterisation of the differential expression of miRNAs in sheep sera

DESeq2^57^ was performed on the sheep miRNA read counts to assess the differential expression of circulating host-miRNAs during infection in comparison to non-infected sheep. The sequencing data from Set A, infection cohort (2, 9, 14, and 18 days post infection; dpi) were grouped as “pre-hepatic infection”, and samples in the Set B cohort (3, 7, 10, 14 weeks post infection; wpi) were grouped as “hepatic infection”, while the sequencing data from 0 dpi (pre-infection) and 14 days non-infected animals were grouped to provide a duplicate set of “non-infected” samples. Only miRNAs with a sum of > 10 counts across all samples and with an adjusted p-value < 0.05 were included in the subsequent analyses. Log2Fold change of 2 (Log2FC2) or fourfold cut-off was applied to identify the most differentially expressed miRNAs.

### RT-qPCR

Custom Taqman small RNA primers were designed for sheep and *F. hepatica* miRNAs, as listed in Table 2. The custom Taqman RT primers and TaqMan™ MicroRNA Reverse Transcription Kit (Applied Biosystems, US) were used for the synthesis of cDNA from 150 ng of RNA extracted from each sample (Set A and B were pooled RNA samples; Set C and D were individual samples), as per manufacturer’s instructions. Then 4.5 μl of cDNA was used for qPCR, with undiluted cDNA used for *F. hepatica* miRNAs, while for the sheep miRNAs the cDNA was diluted 1:2 in dH_2_O. The qPCR was performed using the custom TaqMan primer and TaqMan™ Fast Advanced Master Mix. Samples were prepared on a MicroAmp optical 96-well reaction plate as technical triplicates (Life Technologies, USA) and analysed using the QuantStudio 6 Flex real-time PCR system (Life Technologies, USA). Default settings on the instrument software for Taqman® reagent Fast was used for qPCR reaction, initial denaturation at 95 °C for 20 s, followed by 40 cycles of 1 s denaturation at 95 °C and a 20 s annealing/extension at 60 °C. The starting concentration of the samples, presented as N0 value, was calculated from the qPCR amplification raw data using LinRegPCR software v11^58^.

**Table 2:** Mature miRNA sequences used to design custom TaqMan primers.

Species	miRNA	Canonical/isomiR	Sequence
*Ovis aries*	oar-miR-133-5p	Canonical	UUGGUCCCCUUCAACCAGCUGU
	oar-miR-323a-3p	Canonical	CACAUUACACGGUCGACCUCU
	oar-miR-3957-5p	Canonical	CUCGGAGAGUGGAGCUGUGGGUGU
	oar-miR-1197-3p	Canonical	CCCUUCCUGGUAUUUGAAGACG
*F. hepatica*	fhe-Novel-11-5p	Canonical	AAGCUCGUAGUUGGAUCUGGGU
	fhe-miR-124-3p	Canonical	UUAAGGCACGCGGUGAAUGUCA
	fhe-miR-124-3p	IsomiR	UUAAGGCACGCGGUGAAU

### Statistical analysis

Statistical analysis of RT-qPCR data was performed with GraphPad Prism (v 10). Non-parametric Welch’s two tailed t-test was performed to compare infected versus non-infected samples at each timepoint. *p* value < 0.05 was considered as statistically significant.

## Results

### Differential abundance of host circulating miRNAs during *F. hepatica* infection

To identify the presence of circulating miRNAs that are associated with developing fasciolosis, that may be considered as candidate diagnostic biomarkers, small RNA sequencing was performed on RNA extracted from sera of experimentally infected sheep. Sera collected from two distinct *F. hepatica* infection studies (Set A and B; Table 1) was used for this analysis; Sera in the Set A samples were collected at 2, 9, 14, 18 dpi, and Set B comprised of sera taken at 3, 7, 10, 14 wpi^47^. Sera was also collected from uninfected animals in each study, harvested at time points coincident with a pre-infection (0 dpi,Set A) and 14 weeks (Set B) timepoint.

**Figure 1:**
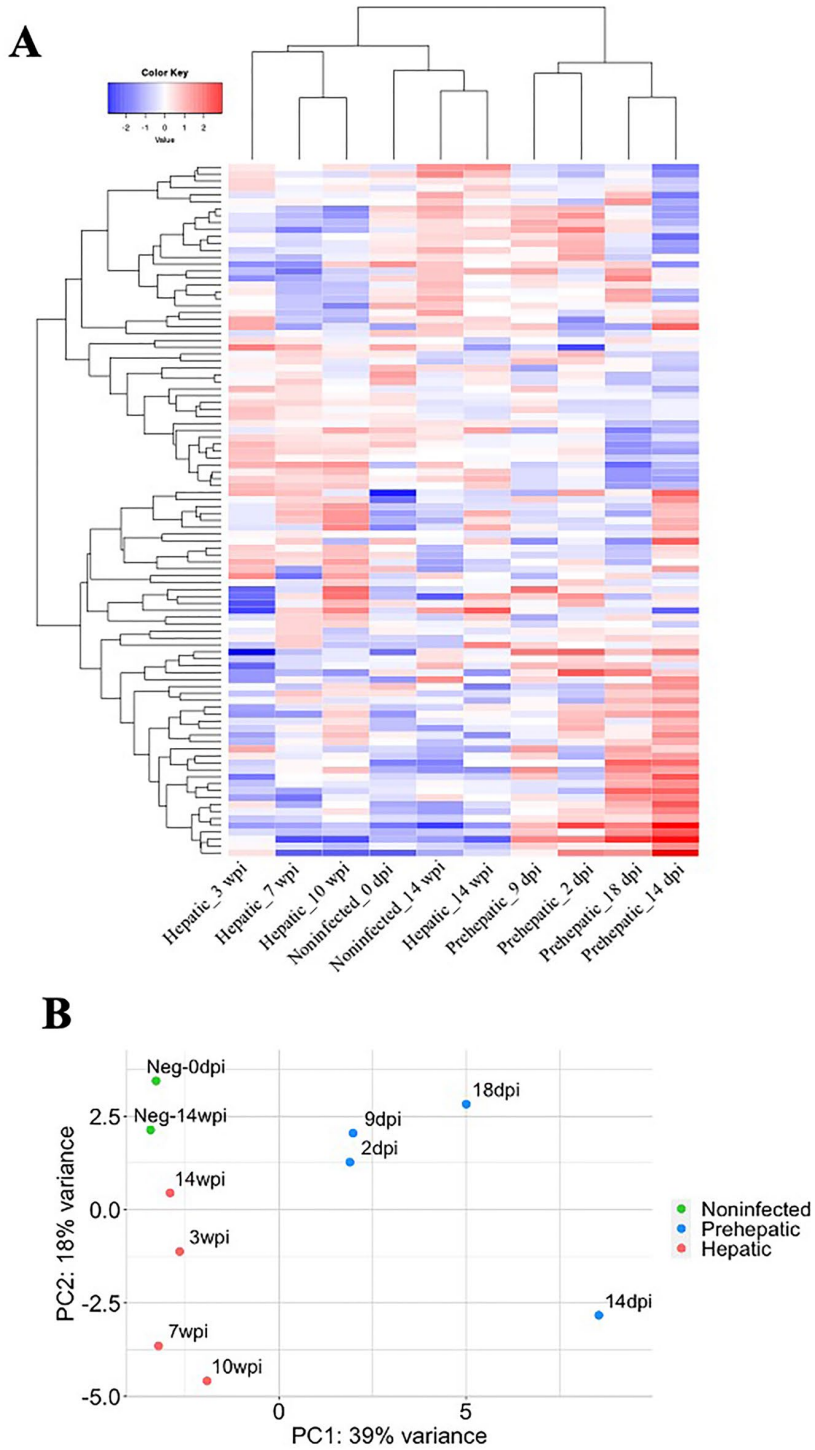
Differential abundance of sheep miRNAs in sera during *Fasciola hepatica* infection. (**A**) Heatmap of relative abundance of sheep miRNAs (CPM) in non-infected sheep (0 day-Neg, 14 week-Neg), early/pre-hepatic infection (2 days post infection (dpi), 9 dpi, 14 dpi, 18 dpi), and late/hepatic infection (3 weeks post infection (wpi), 7 wpi, 10 wpi, 14 wpi). The heatmap was created using idep.96^59^, miRNAs were ranked by their standard deviation across all samples and hierarchical clustering carried out for the top 100 miRNAs . Expression of miRNAs represented as high (red) or low (blue) relative to total expression of miRNA across infection groups. (**B**) Principal component analysis (PCA) plot of total sheep miRNA abundance in non-infected sheep (green), pre-hepatic infection (2–18 days post infection) (blue), and hepatic infection (3–14 weeks post infection) (red).

As the initial RNA sequencing analysis of these individual samples was unsuccessful due to low RNA yield, RNA from six sera samples at each time point was pooled for preparation of the small RNASeq library. The resulting sequencing data was screened against the 153 mature miRNA sheep sequences registered in miRBase (Oar_V4.0). On average, 104 and 108 out of 153 sheep miRNAs were detected in the uninfected and infected sheep samples, respectively (Supplemental Table 2). Hierarchal clustering of the sequencing data from the different time points revealed a distinct separation in the miRNA expression between early and late infection (Fig. 1A). To measure differential expression (DE) during disease progression, the sequencing reads were grouped as pre-hepatic (2, 9, 14 and 18 dpi; thus n = 4) and hepatic (3, 7, 10 and 14 wpi; n = 4) samples. Comparative analysis was then conducted using the expression profile from the sera from non-infected sheep (pre-infection 0 dpi and 14 wpi; n = 2). Principal component analysis (PCA) verified that samples produced distinct clusters, correctly grouping the pre-hepatic and hepatic samples (Fig. 1B).

Comparing the sheep miRNAs between the non-infected, pre-hepatic, and hepatic stages of infection revealed several distinct miRNAs that were significantly increased or decreased in their abundance during the progression of infection. A four-fold cut-off (Log2FC2) was applied to these miRNA profiles to distinguish the subset of miRNAs that were most highly altered in expression and, therefore, most likely to be above a threshold of differential abundance to support the diagnosis of infection and potentially differentiate between the pre-hepatic and hepatic stages of disease.

**Figure 2:**
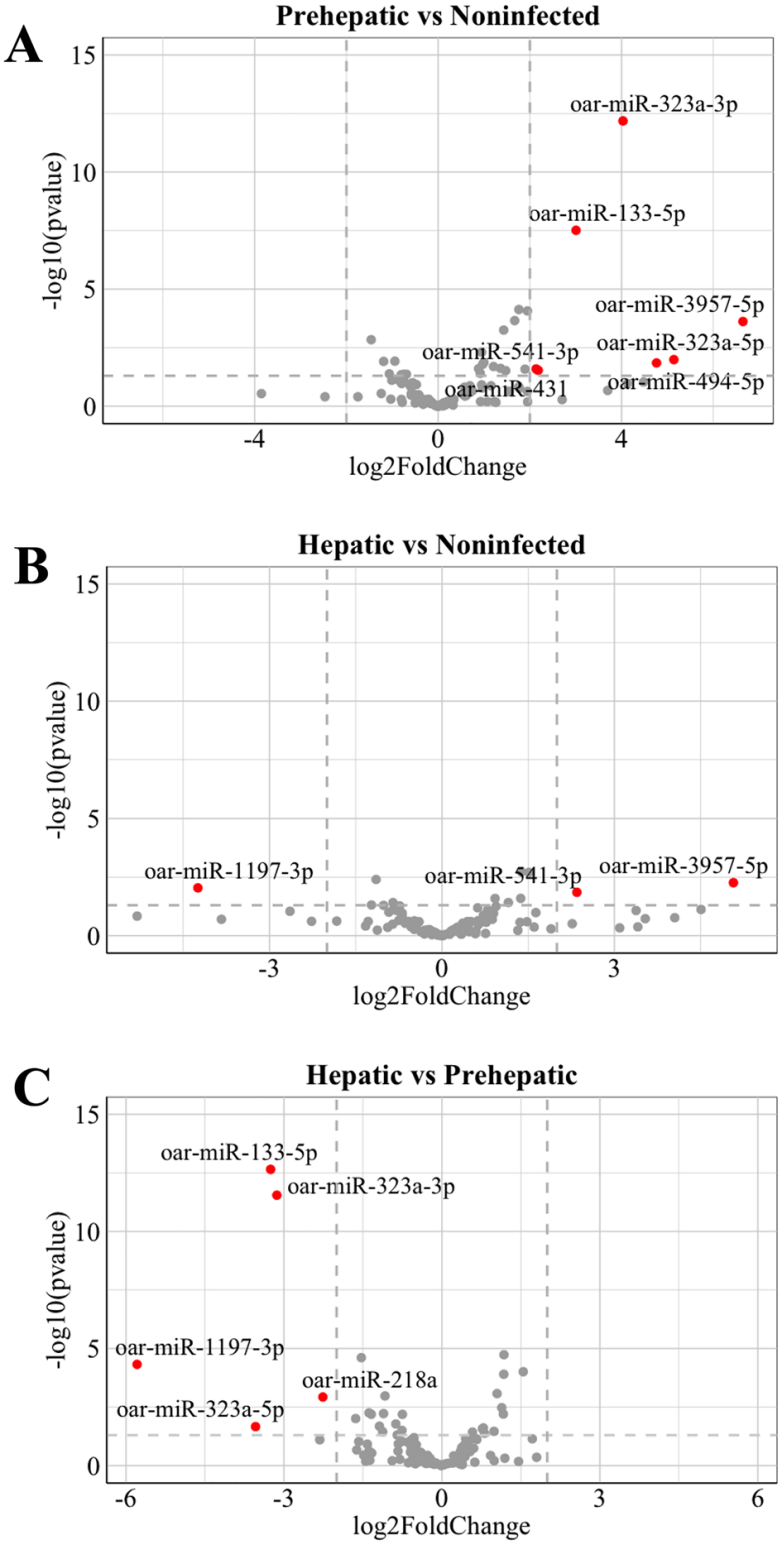
Circulating host-miRNA profiles are distinct during early and late *F. hepatica* infection compared to non-infected animals. Volcano plots show differential expression of sheep miRNAs using DESeq2 in (**A**) pre-hepatic infection compared to non-infected sera samples, (**B**) hepatic infection vs non-infected sera samples, and (**C**) hepatic infection vs pre-hepatic infection. Genes that are more than ± log2 fold change 2 (FC) with significant *p* value (< 0.05) are denoted in red.

This revealed the presence of six miRNAs that significantly increased during the pre-hepatic stage of the infection phase when compared to non-infected animals (Fig. 2A; Supplemental Table 3A). Of these, the change in expression ranged from 2 to sixfold, but the differential expression of oar-miR-323a-3p, oar-miR-133-5p, and oar-miR-3957-5p were the most significantly increased in the infected animals. The increased expression of oar-miR-3957-5p and oar-miR-541-3p, seen in the pre-hepatic samples, was also evident during the hepatic stage in infected sheep as compared to non-infected sheep, albeit at a reduced level (Fig. 2B; Supplemental Table 3B). In contrast, the presence of oar-miR-133-5p, and oar-miR-323a-3p were significantly downregulated during the hepatic stage of infection, as compared to the pre-hepatic timepoints (Fig. 2C; Supplemental Table 3C). Additionally, oar-miR-1197-3p was identified as the only miRNA significantly reduced during the hepatic stage of infection compared to uninfected animals and pre-hepatic infection (Fig. 2B,C; Supplemental Table 3B,C).

### Small RNA sequencing revealed the presence of *F. hepatica* derived miRNAs in the serum of infected sheep

To determine if miRNAs derived from *F. hepatica* were present in the sera from infected sheep, sequencing reads that did not align to sheep mature miRNAs were screened against known *F. hepatica* miRNAs (Supplemental Table 1). Critically, no parasite-derived miRNA sequences were identified within the sera samples collected from non-infected sheep and, thus, validated this approach. In contrast, six liver fluke miRNAs were found within the sera from infected sheep (Table 3). These miRNAs displayed a distinct pattern of expression correlating to the division of samples into pre-hepatic and hepatic phases of infection. Specifically, two miRNAs were detected only at the pre-hepatic stage of infection: fhe-miR-124-3p and fhe-miR-71a-5p; however, fhe-miR-124-3p had higher read counts and was observed at all the timepoints, suggesting it may be a more robust infection marker than fhe-miR-71a-5p. While three microRNAs (fhe-Novel-102-3p, fhe-miR-277a-3p, and fhe-miR-750-3p) were linked to the hepatic phase of the infection, their counts per million (CPM) were all below 13, suggesting a low abundance. Only fhe-Novel-11-5p, was found throughout the entire infection, detected in all serum samples from both the pre-hepatic and hepatic stages.

**Table 3:** Total read counts of *Fasciola hepatica* miRNAs detected within sheep sera samples.

miRNA	Non-infected		Pre-hepatic				Hepatic			
	Neg-0 dpi	Neg-14 wpi	2 dpi	9 dpi	14 dpi	18 dpi	3 wpi	7 wpi	10 wpi	14 wpi
fhe-miR-124-3p	0	0	59	32	44	28	0	0	0	0
fhe-Novel-11-5p	0	0	7	0	0	3	13	12	6	0
fhe-Novel-102-3p	0	0	0	0	0	0	0	13	9	0
fhe-miR-277a-3p	0	0	0	0	0	0	0	8	5	7
fhe-miR-750-3p	0	0	0	0	0	0	0	5	0	4
fhe-miR-71a-5p	0	0	0	8	0	0	0	0	0	0

(n = 6 sheep sera pooled/ time point); *dpi* Days post-infection, *wpi* Weeks post-infection, *Neg-0 dpi* Pre-infection timepoint, *Neg-14 wpi* 14 Weeks age-matched non-infected control.

### Selection of sheep and *F. hepatica* derived miRNAs as potential biomarkers of fasciolosis

**Figure 3:**
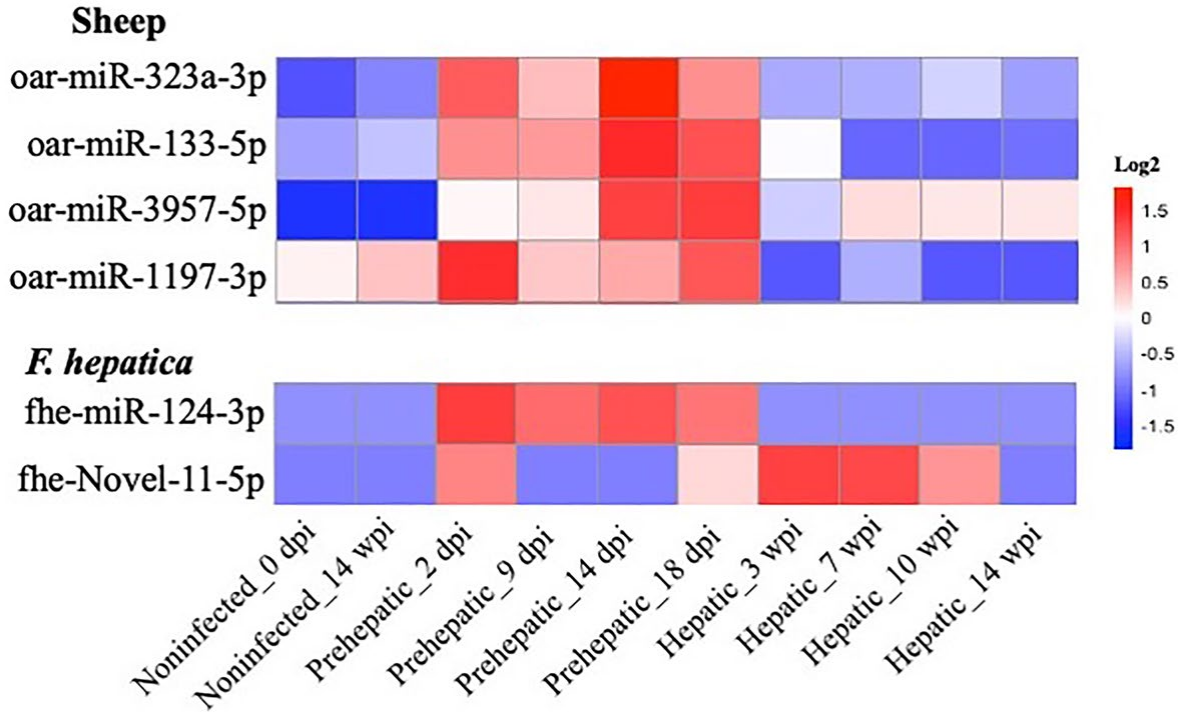
Selection of host and *F. hepatica* miRNAs as biomarkers for pre-hepatic and hepatic stages of fasciolosis. Relative abundance of selected sheep and parasite miRNAs in uninfected animals (0 dpi-neg, 14 wpi-neg), pre-hepatic infection (2 dpi, 9 dpi, 14 dpi, 18 dpi), and hepatic infection (3 wpi, 7 wpi, 10 wpi, 14 wpi), represented as high (red) or low (blue) normalised read counts. *dpi* Days post infection, *wpi* Weeks post infection.

Based on our sequence analysis, a panel of sheep and parasite-derived miRNAs were curated as potential biomarkers of *F. hepatica* infection due to their differential abundance in infected compared to non-infected sheep and their presence across the different stages of infection (Fig. 3). This panel comprised four sheep miRNAs and two *F. hepatica* miRNAs. The sheep miRNAs included oar-miR-133-5p and oar-miR-323a-3p, which were the most significantly upregulated miRNAs in pre-hepatic infection versus non-infected animals; oar-miR-3957-5p which was consistently elevated (log2FC4) in pre-hepatic and hepatic infection; and oar-miR-1197-3p which was downregulated in hepatic infection compared to both non-infected and pre-hepatic infection. The parasite miRNAs for the selected biomarker panel included fhe-miR-124-3p and fhe-novel-11-5p, which had the highest read numbers in sheep sera.

While sequencing analysis provided high-throughput screening of RNA sequences, it is not practical for large-scale diagnosis of fasciolosis. RT-qPCR represents a more suitable method for determining the differential expression of miRNAs in simultaneous samples of sheep sera. On this basis, a series of qPCR primers specific to each of the selected miRNA sequences was designed. To first test the ability of these primers to specifically detect the candidate diagnostic miRNAs, lamb tissue was employed as a positive test sample for evaluating the performance of sheep miRNA specific primers, while RNA isolated from *F. hepatica* NEJ was used as the positive control to assess the efficacy of primers designed to detect the liver fluke miRNAs. This life cycle stage (NEJ) of the parasite was selected because fhe-Novel-11-5p is reportedly only expressed in NEJ and not in the juvenile or adult worms (both liver stages), while fhe-miR-124-3p is present throughout all intra-mammalian life cycle stages with the highest expression in the NEJ^45^. Based on the results from these RT-qPCR tests, oar-miR-1197-3p was excluded from further analysis due to its low copy number and late Cq (~ 33) in lamb tissue, with undetectable levels in non-infected sheep sera (Supplemental Table 4). According to the sequencing data, the expression level of this miRNA was expected to decrease during the hepatic phase of infection (Fig. 3). However, as it was not amplified in the test sera sample from non-infected sheep, a further decrease following the progression of *F. hepatica* infection would be impossible to detect by qPCR, thus negating its potential as a biomarker for hepatic infection.

In contrast, the Cq value of oar-miR-133-5p, oar-miR-323a-3p, and oar-miR-3957-5p were within an acceptable diagnostic range (Supplemental Table 4), making them suitable for further analysis. In addition, the amplification of products from the Set A and B sera samples using these primers validated the sequencing data, as the pattern of expression was consistent with the distribution of read counts within the sequencing data. This RT-qPCR analysis verified that both oar-miR-133-5p and oar-miR-323a-3p were only present in the pre-hepatic set of samples whereas oar-miR-3957-5p was detected in samples from both the pre-hepatic and hepatic stages of infection (Supplemental Fig. 1).

Of the *F. hepatica* miRNAs, fhe-miR-124-3p and fhe-Novel-11-5p primers unexpectedly amplified products in both NEJ and lamb tissue (Supplemental Figs. 2A, 3A). Also, the primers designed to detect fhe-miR-124-3p produced extremely late, or no Cq, when tested further on the RNA samples from the sera from infected sheep that had been sequenced (Sets A and B; Supplemental Fig. 2B). Given these inconsistencies with the results from the test RT-qPCR in comparison to the sequencing data, a more in-depth analysis of the sequencing data for these two miRNA sequences was conducted.

This analysis revealed that the full-length canonical sequence of fhe-Novel-11-5p was not present within the sequencing reads, but a shorter variant (isomiR) missing four nucleotides at the 3′ end was identified instead. Furthermore, by aligning this shorter sequence to the known sheep sequences using BLASTN (filtering for *Ovis aries*, txid:9940) revealed that the fhe-Novel-11-5p sequence fully aligned with sheep ribosomal RNA (Supplemental Fig. 3B), which provides an explanation for the detection of fhe-Novel-11-5p in lamb tissue. In addition, as ribosomal RNAs are part (10%) of the extracellular RNA population found in sera due to cellular turnover or secretion of microvesicles as a consequence of tissue injury^60^, it is likely that the detection of this sequence in the sera of infected sheep, but not uninfected sheep, reflects the host response to infection with Fasciola, or the tissue damage caused by the migrating parasite.

However, due to the uncertainty of whether this result is unique to *F. hepatica* and thus indicative of infection, fhe-Novel-11-5p was omitted from subsequent analyses.

**Figure 4:**
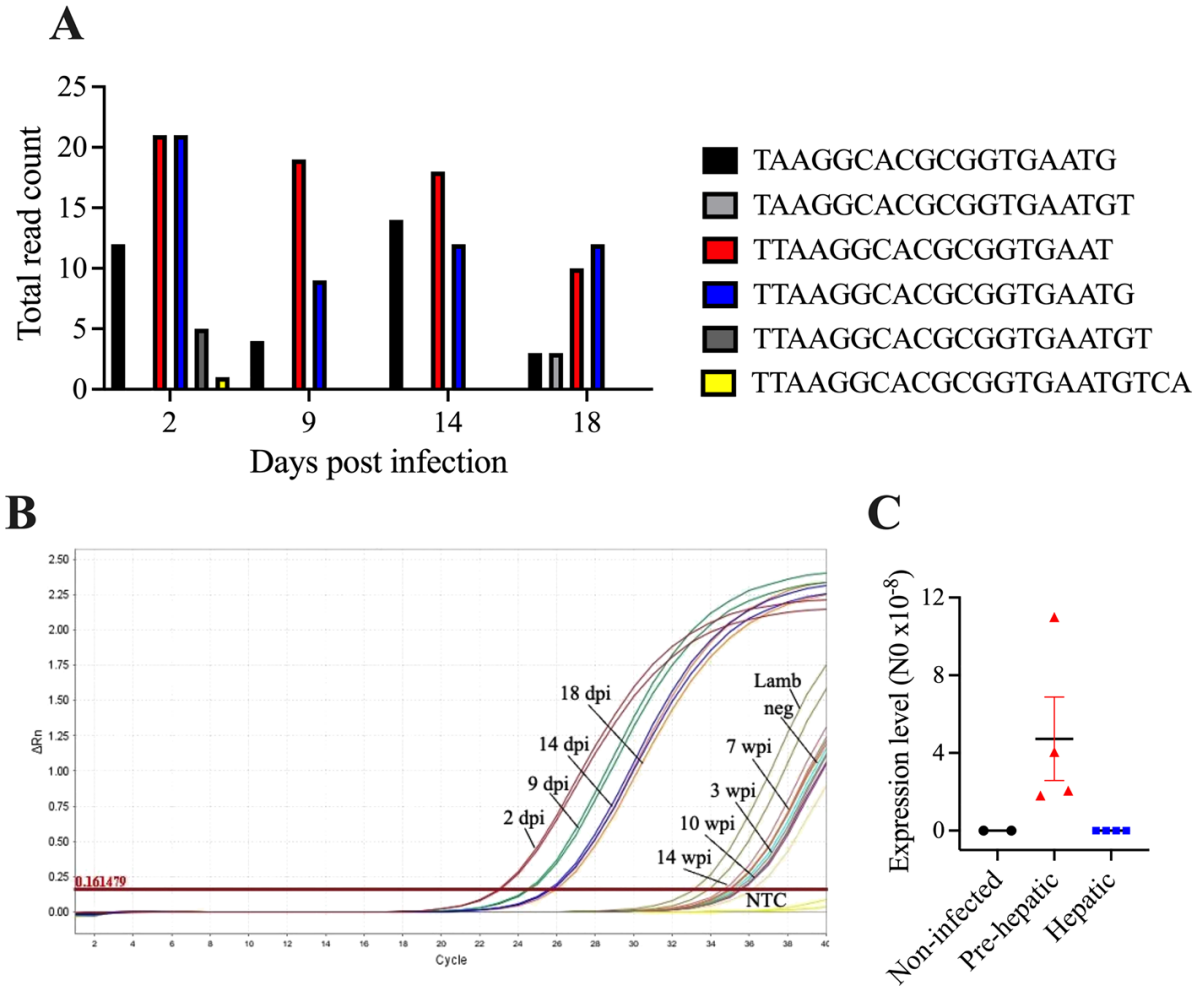
*F. hepatica* miR-124-3p was present as several sequence variants (isomiR) in sera of infected sheep. (**A**) Total read counts of the miR-124 isomiRs present at the different time-points of infection in Set A and Set B sera. The canonical sequence is shown in yellow. The sequence of the most dominant isomiR (red bar) was used for qPCR primer design. (**B**) RT-qPCR amplification plot from primer test of the most dominant fhe-miR-124 isomiR in Set A and Set B sheep sera samples. *dpi* Days post infection, *wpi* Weeks post infection, *neg* Non-infected controls, *NTC* No template control and lamb tissue. (**C**) RT-qPCR quantification of the dominant fhe-miR-124-3p isomiR presented as the starting quantity of genetic material prior to amplification (N0) as determined by LinRegPCR (v.11) This is calculated in the unit of the Y-axis of the PCR amplification plot, which are arbitrary fluorescence units. The average N0 value of two technical replicates are shown for each time point.

While the canonical sequence for fhe-miR-124-3p was not present in the sequencing reads from the sheep sera samples, this miRNA was found to be present as several sequence variants (isomiRs), missing nucleotides primarily at the 3′ end and to a lesser extent at the 5′ end (Fig. 4A). Furthermore, the sequence of the canonical fhe-miR-124-3p was similar to cattle bta-miR-124-3p (Supplemental Fig. 4A), with only three nucleotides mismatched at the 3′ end, suggesting that the corresponding sheep oar-miR-124-3p was likely missing from miRbase, and was thus not filtered from the sequencing reads during our initial analysis.

Consequently, a 100% sequence identity of the bta-miR-124-3p precursor sequence to the non-coding region of sheep RNA confirmed the presence of oar-miR-124-3p and that oar-miR-124-3p is identical to bta-miR-124-3p (Supplemental Fig. 4B). Accordingly, the shorter two most dominant sequence variants of fhe-miR-124-3p found in infected sheep sera matched 100% with both sheep and *F. hepatica* miR-124-3p (Fig. 4A). Therefore, it is impossible to determine whether the sequencing reads for the miR-124-3p variant that are increased during *F. hepatica* infection originated from sheep, parasite, or both. Nonetheless, the differential abundance of this miRNA classified it as a suitable candidate as a potential biomarker of infection and, thus, a primer was designed for the most dominant 3′ trimmed variant of miR-124-3p. This primer effectively distinguished the pre-hepatic stage of infection (Cq < 26) from both the hepatic stage and non-infected (Cq > 33) sheep using the Set A and B sequenced sera samples, an outcome that was consistent with the read counts from the sequencing data (Fig. 4B,C; Table 3).

Based on these analyses, the miRNA biomarker panel for qPCR validation was refined to include four miRNAs: oar-miR-133-5p, oar-miR-323a-3p, oar-miR-3957-5p, and fhe/oar-miR-124-3p.

### Circulating oar-miR-133-5p and oar-miR-3957-5p can diagnose fasciolosis from as early as 7 days post-infection

To confirm the diagnostic potential of the selected miRNAs, RT-qPCR validation was performed on an independent archived set of sera samples harvested from individual sheep during two longitudinal infection studies; a pre-hepatic infection cohort comprised of sera collected at 0 (pre-infection), 7, and 14 days post-infection (Set C), a hepatic infection cohort with samples collected at week 3, 15, 20 and 23 post-infection (Set D), and age-matched non-infected animals for each of the timepoints.

**Figure 5:**
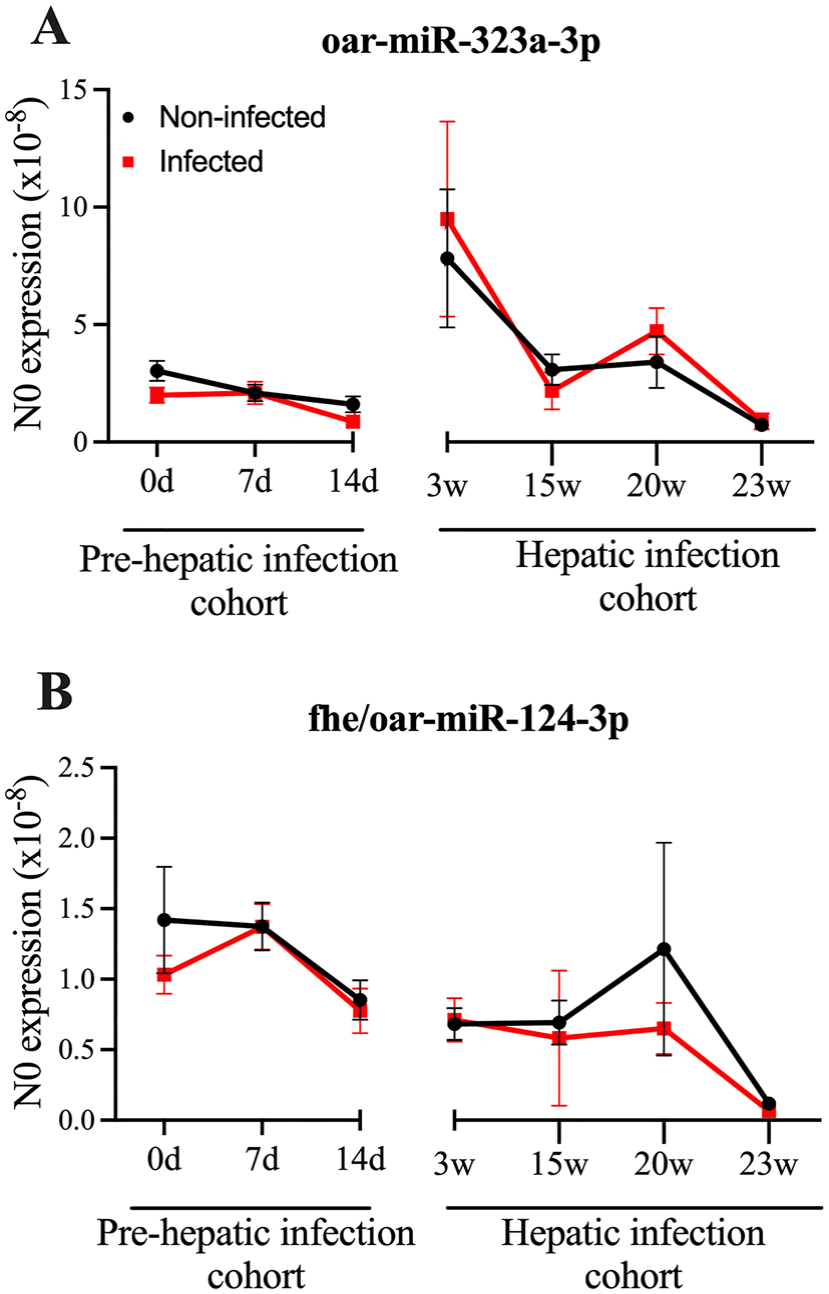
RT-qPCR assessment of sera samples invalidates the diagnostic potential of oar-miR-323a-3p and fhe-miR-124-3p. The expression of (**A**) oar-miR-323a-3p, (**B**) fhe-miR-124-3p were determined by RT-qPCR and are presented as the starting quantity of genetic material prior to amplification (N0) as determined by LinRegPCR (v.11). This is calculated in the unit of the Y-axis of the PCR amplification plot, which are arbitrary fluorescence units. The data is presented as the mean ± SEM (n = 3–14; as shown in Table 1). The statistical significance of differences between infected and non-infected samples was determined using Welch’s t-test (two-tailed).

From this analysis, the expression levels of both oar-miR-323a-3p and fhe/oar-miR-124-3p was almost identical in the sera from non-infected and infected sheep over the course of infection (Fig. 5A,B), suggesting that these miRNAs cannot be used as indicators of fasciolosis. It is possible that this discrepancy between this RT-qPCR analysis and the sequencing data reflects the difference in sample preparation, with the sequencing performed on a pooled sample of sera from a cohort of six sheep. The variability in the expression of miRNAs across sheep samples is evident when sera from individual sheep was assessed by RT-qPCR, which in a pooled sample would create an incorrect representation of expression levels within individual samples. Furthermore, through the inclusion of age-matched non-infected sheep, it is apparent that the expression of miRNAs can change over time, irrespective of whether the animal is infected (Fig. 5). As the sample collection that was originally sequenced lacked a full panel of age-matched controls, this alteration in expression was not fully captured, and the apparent differential abundance of miRNAs associated with infection within the sequencing data, may have simply been a variation due to age.

**Figure 6:**
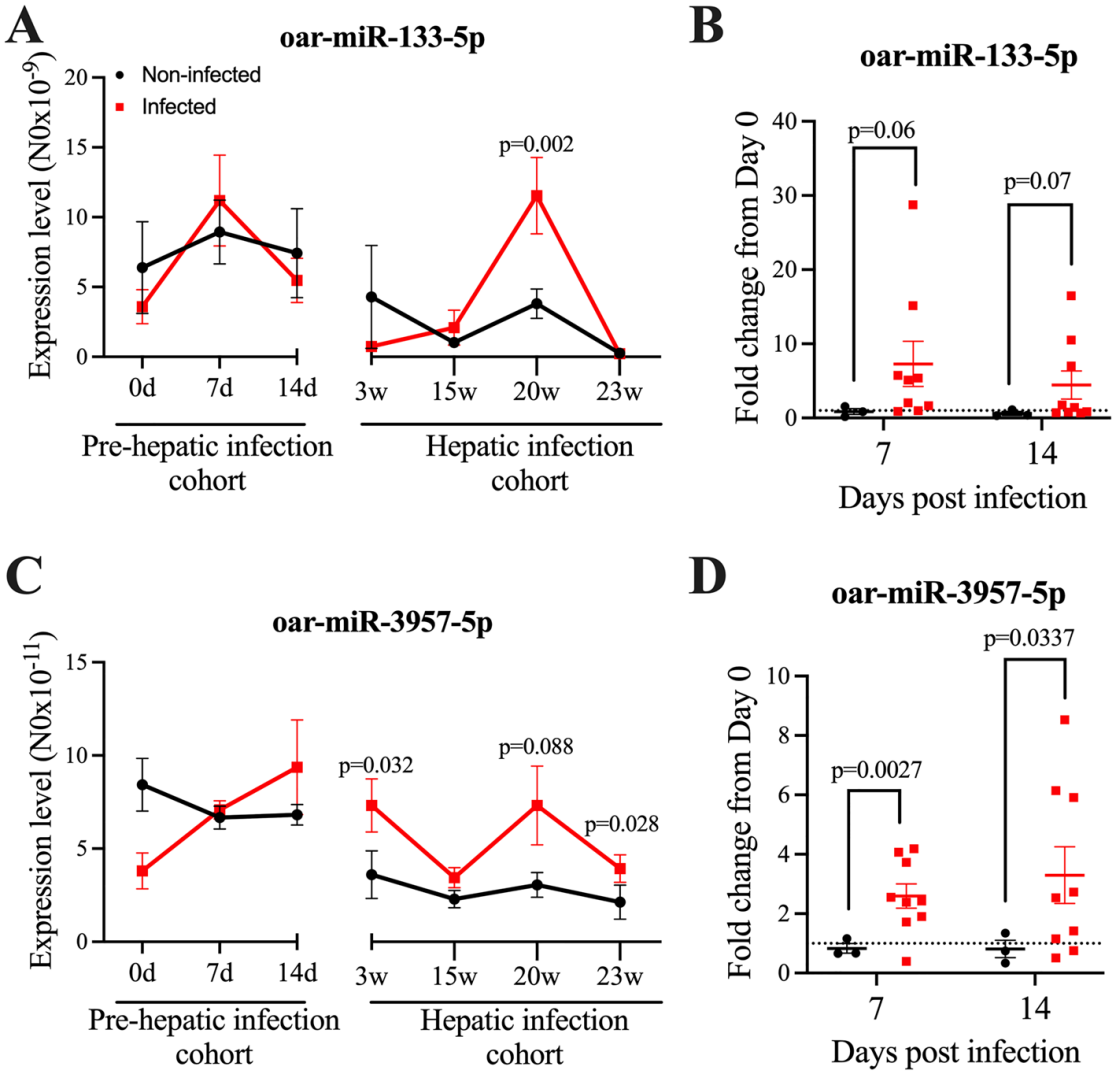
RT-qPCR demonstrates the differential expression of oar-miR-133-5p and oar-miR-3957-5p in sera from infected sheep as compared to uninfected animals. Quantification of (**A**) oar-miR-133-5p and (**C**) oar-miR-3957-5p in the sera of individual sheep in response to infection was determined by RT-qPCR and is represented as the starting quantity of genetic material prior to amplification (N0) as determined by LinRegPCR (v.11). This is calculated in the unit of the Y-axis of the PCR amplification plot, which are arbitrary fluorescence units. The data is presented as the mean ± SEM (n = 3–14; as shown in Table 1). (**B**) Fold change of oar-miR-133-5p and (**D**) oar-miR-3957 expression at 7 or 14 dpi in comparison to 0 dpi in the sera of individual sheep in the pre-hepatic cohort. The data is presented as the mean ± SEM (n = 3 non-infected; 9 infected sheep for which data was available at all three timepoints). In all cases the statistical significance of differences between sera from infected versus uninfected sheep was determined using Welch’s t-test (two-tailed).

Consistent with the sequencing data, the RT-qPCR analysis revealed that the expression of both oar-miR-133-5p and oar-miR-3957-5p was elevated in the sera of infected animals compared to age-matched non-infected animals (Fig. 6A,C). Furthermore, as these were longitudinal studies, the miRNA expression level in individual sheep in the pre-hepatic infection cohort could be tracked over time. As such, the fold change at 7 and 14 days compared to day 0 for each animal was examined. This approach revealed that the expression levels of both oar-miR-133-5p and oar-miR-3957-5p were significantly elevated in the infected animals at day 7 and 14 from day 0, but not in the non-infected animals (Fig. 6B,D). Early elevation in the levels of these two miRNAs was consistent with the sequencing data, whereby an upregulation in miRNA expression was seen from 2 dpi (Fig. 3). The sera samples representing the hepatic stage of infection were harvested from a separate infection cohort to the earlier time points, so could not be tracked in individual sheep from day 0.

Nonetheless, corroborating the sequencing data, the level of expression of oar-miR-133-5p in the sera of infected animals, other than a single peak at 20 wpi, was reduced as infection progressed, as compared to the earlier time points (Fig. 6A). In addition, validating the sequencing data, the increase in oar-miR-3957-5p in sera from infected sheep was generally sustained throughout the entire observation period, with a lower expression level compared to the pre-hepatic phase of infection (Fig. 6C).

In conclusion, these data indicate that both oar-miR-133-5p and oar-miR-3957-5p are biomarkers of early, pre-hepatic infection. In addition, the difference in expression of oar-miR-3957-5p between infected and non-infected sheep over the entire observation period suggests that this may represent an unchanging marker of infection, which, in the absence of an increase in oar-miR-133-5p, would indicate late stage/hepatic phase of infection.

## Discussion

The severe impact of fasciolosis on animal welfare and the significant economic cost it imposes on the livestock industry highlights the need for a reliable and robust biomarker that can effectively guide anthelmintic treatment and facilitate the management and control of liver fluke on farms^17^. The development of an accurate diagnostic method for acute infection is of paramount importance since such a tool would enable timely intervention to prevent liver flukes from inflicting liver pathology, which would mitigate the risk of sudden fatalities associated with a high liver fluke burden (in approximately 10% of infected sheep) and disrupt the liver fluke lifecycle by preventing the parasite from reaching the bile duct to lay eggs, which in turn, would curb the spread of the infection within grazing pastures.

Given this need, exploiting the differential abundance of circulating miRNAs as a biomarker of infection represents an attractive diagnostic tool. Circulating miRNAs exhibit exceptional stability, rendering them a highly suitable choice for diagnostic purposes, particularly in the setting of a farm where controlling sample collection and storage can be challenging. For this reason, miRNAs have gained significant attention for their diagnostic potential in a range of pathogenic infections, and due to their immediate alteration in expression in response to infection they are appropriate markers for the detection of early infections^39^.

In this study, we assessed the utility of miRNAs in detecting both pre-hepatic and hepatic fasciolosis using sera samples collected from sheep with experimental subclinical disease. The results provide the first evidence that unique miRNA expression signatures in ruminant sera are associated with the distinct pathological stages of fasciolosis. The data supports the hypothesis that differentially expressed miRNAs can serve as prognostic markers for fasciolosis and, moreover, demonstrates that unique miRNA expression patterns emerged as early as 2 days post-infection. Therefore, miRNAs could offer superior sensitivity in fasciolosis diagnosis compared to conventional, commercial methods, such as FEC and serological ELISAs.

The analysis of sera samples from age-matched infected and non-infected sheep in the RT-qPCR validation demonstrated the paramount importance of incorporating age-matched samples in any screening protocol aimed at characterising miRNAs as diagnostic biomarkers. As demonstrated in this study, the differential expression of miRNAs quantified using RT-qPCR exhibited significant age-related variability. Specifically, the expression of oar-miR-323a-3p, which we initially described as a potential biomarker from sequencing data (without appropriate age-matched samples), was subsequently discovered using RT-qPCR to vary significantly over time in non-infected sheep, with relatively higher expression as the animals aged.

Despite encountering challenges with variations in miRNA expression levels due to age, our longitudinal approach of assessing miRNA expression in individual animals over time successfully addressed these issues. Our findings indicate that the expression of certain miRNAs in sheep increased in response to infection, irrespective of the animal's age. Given this observation, we would recommend that for any miRNA-based diagnostics studies, it may be necessary to collect serum samples from animals at their initial exposure to pasture to set a benchmark for each animal's miRNA expression. Future serum samples can then be compared to this baseline to accurately assess the onset and progress of infection. This approach could also be effectively adapted to sentinel screening practices for monitoring liver fluke prevalence over a grazing season, as we have employed with serological tests^61,62^.

While the potential of using miRNA detection to support the diagnosis of fasciolosis has been established in this study, further evaluation is required to ensure specificity. For example, circulating oar-miR-3957-5p has been reported to be elevated in high stress responding female sheep when challenged with bacterial LPS^63,64^ suggesting that this miRNA could be upregulated in response to other broad infections. Furthermore, the sheep in our study were all male; the possibility that any differential expression in miRNAs is sex-related would need to be considered. In addition, miR-133-5p was reportedly upregulated in early bacterial infection in a marine lancelet species (*Branchiostoma belcheri*)^65^, and in the plasma of chronic obstructive pulmonary disease patients, and tumour cells of various cancers, including gastric and colon cancer^66,67^. Hence, while oar-miR-133-5p and oar-miR-3957 are indicated as promising biomarkers for fasciolosis, their specificity to fasciolosis must be further explored.

To address the issue of specificity in another way, the results of differential expression of host miRNAs could be amalgamated with the detection of liver fluke miRNAs. Liver fluke miRNAs are likely deliberately secreted by the parasite to manipulate the host's immune response or are passively released from dying parasites. While several helminth studies have demonstrated the presence of parasite-derived miRNAs in host circulation^42,43,68^, this is the first study to demonstrate the presence of *F. hepatica* derived miRNAs in infected host circulation (importantly that of a ruminant). Furthermore, the temporal expression of the liver fluke-derived miRNAs correlated with their expression pattern that we previously reported over the course of the intra-mammalian stages of development^45^. For instance, fhe-miR-124-3p and fhe-miR-71a-5p, both of which were found to be most abundant in NEJ, were detected in sheep sera at the pre-hepatic stage of infection. On the other hand, fhe-miR750-3p and fhe-Novel-102-3p, both most highly expressed by adult worms, were detected at the hepatic stage of infection. Notwithstanding that many parasite-derived miRNAs are present at very low copy numbers, it remains to be determined whether they could be amplified to detectable levels of RT-qPCR.

We also discovered that several helminth miRNAs are highly conserved and, therefore, have sequences that are nearly identical to their mammalian hosts. In this study, *F. hepatica*-derived miRNAs in sheep sera were detected using a rigorous filtering process in the analysis of sequencing reads; only reads that did not align with the sheep mature miRNAs from miRBase were aligned to *F. hepatica* miRNAs, allowing zero mismatches, to eliminate any false identification of sheep miRNAs as parasite miRNAs. Furthermore, the presence of the parasite-miRNAs in sera samples from infected sheep but not in the non-infected samples provided confidence that these miRNAs exclusively originated from the parasite. However, further investigation revealed that two of the identified liver fluke miRNAs, namely fhe-miR-124-3p and fhe-Novel-11-5p, were present as trimmed isomiRs in the sheep sera, that were also conserved with sheep sequences. While previous studies have reported the presence of helminth-derived miRNAs in host circulation^42,43,68^, the existence of such sequence variants has not yet been documented. This unique discovery raises important new considerations regarding the interpretation of sequencing data for the discovery of helminth (or pathogen)-derived miRNAs such as miR-124-3p.

In summary, our research has characterised the differential abundance of parasite and host serum miRNAs during infection with *F. hepatica* in a ruminant host. Two miRNA biomarkers with diagnostic potential for fasciolosis were identified following a stringent selection process. Lowering the threshold of selection from sequencing data to a twofold change in expression could reveal additional miRNA targets and provides an opportunity to expand the available miRNA biomarker panel. The data presented here has established the suitability of developing the measurement of sheep miRNA expression into a diagnostic tool for fasciolosis, potentially through a PCR blood test. Achieving this potential will require extensive field studies to determine the precise sensitivity and specificity of this method. While our current findings are based on controlled experimental infections of sheep, the ultimate goal will be to test the effectiveness of these miRNA biomarkers in naturally infected animals. Such real-world conditions present a more complex scenario for diagnosis due to varying worm burdens, infection dynamics, overlapping temporal infections, and the potential for co-infections (liver fluke and other pathogens). Importantly, validation of these miRNA biomarkers in the field could lead to their use in point-of-care technologies at the farming level. This would be particularly beneficial in environments where access to technical expertise and resources is limited, offering a practical and user-friendly method for detecting fasciolosis in the field.

### Supplementary Information

Supplementary Figures.

Supplementary Table S1.

Supplementary Table S2.

Supplementary Table S3.

Supplementary Table S4.

## Data Availability

The RNA sequencing data have been deposited in NCBI’s Gene Expression Omnibus and are accessible through GEO Series accession number GSE254115. All other data is available from the corresponding author on reasonable request.
