# Supplementary material for: Exploring the utility of circulating miRNAs as diagnostic biomarkers of fasciolosis
Source: Sci Rep. 2024 Mar 28;14:7431. doi: 10.1038/s41598-024-57704-9 (PMC10978983; doi:10.1038/s41598-024-57704-9)
Supplement: Supplementary file 1 — Supplementary Figures. [file 41598_2024_57704_MOESM1_ESM.pdf]

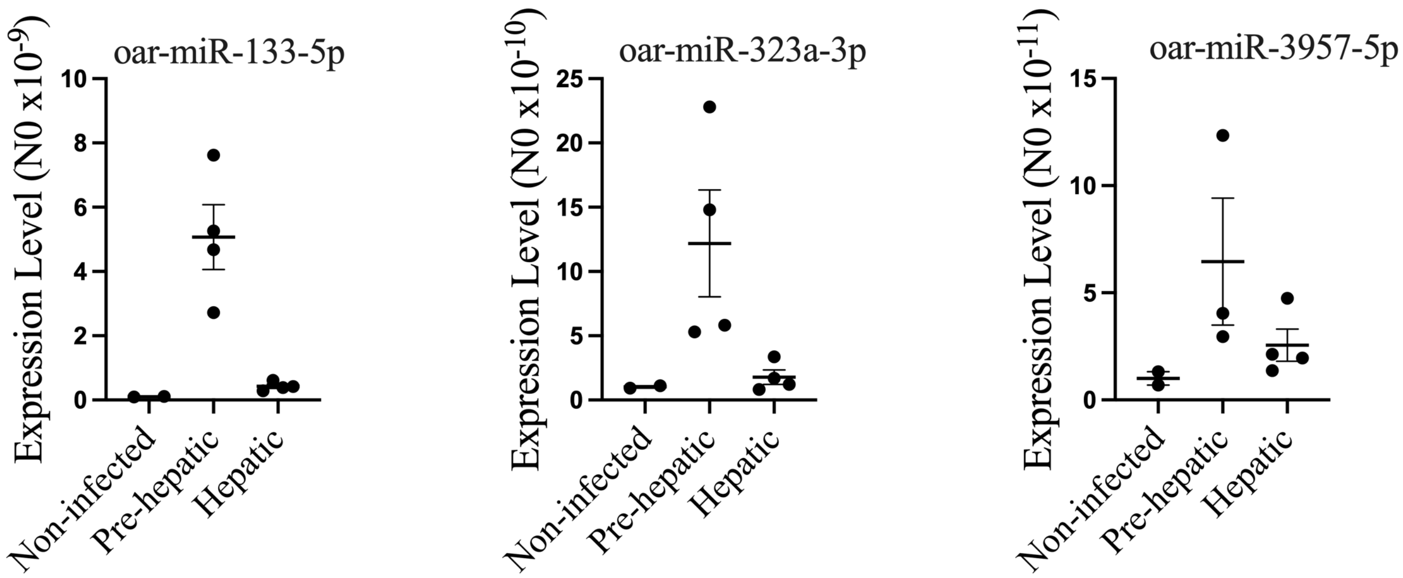


**Supplementary Figure 1. Validation of sequencing data using PCR and primers designed to detect the expression of sheep miRNAs**. RT-qPCR quantification of oar-miR-133-5p, oar-miR-323a-3p and oar-miR-2957-5p expression in the RNA isolated from the Set A and Set B sheep sera samples that had previously been sequenced. The data is presented as the starting quantity of genetic material prior to amplification (N0) as determined by LinRegPCR (v.11) This is calculated in the unit of the Y-axis of the PCR amplification plot, which are arbitrary fluorescence units.  The average N0 value of two technical replicates are shown for each time point.

A

B

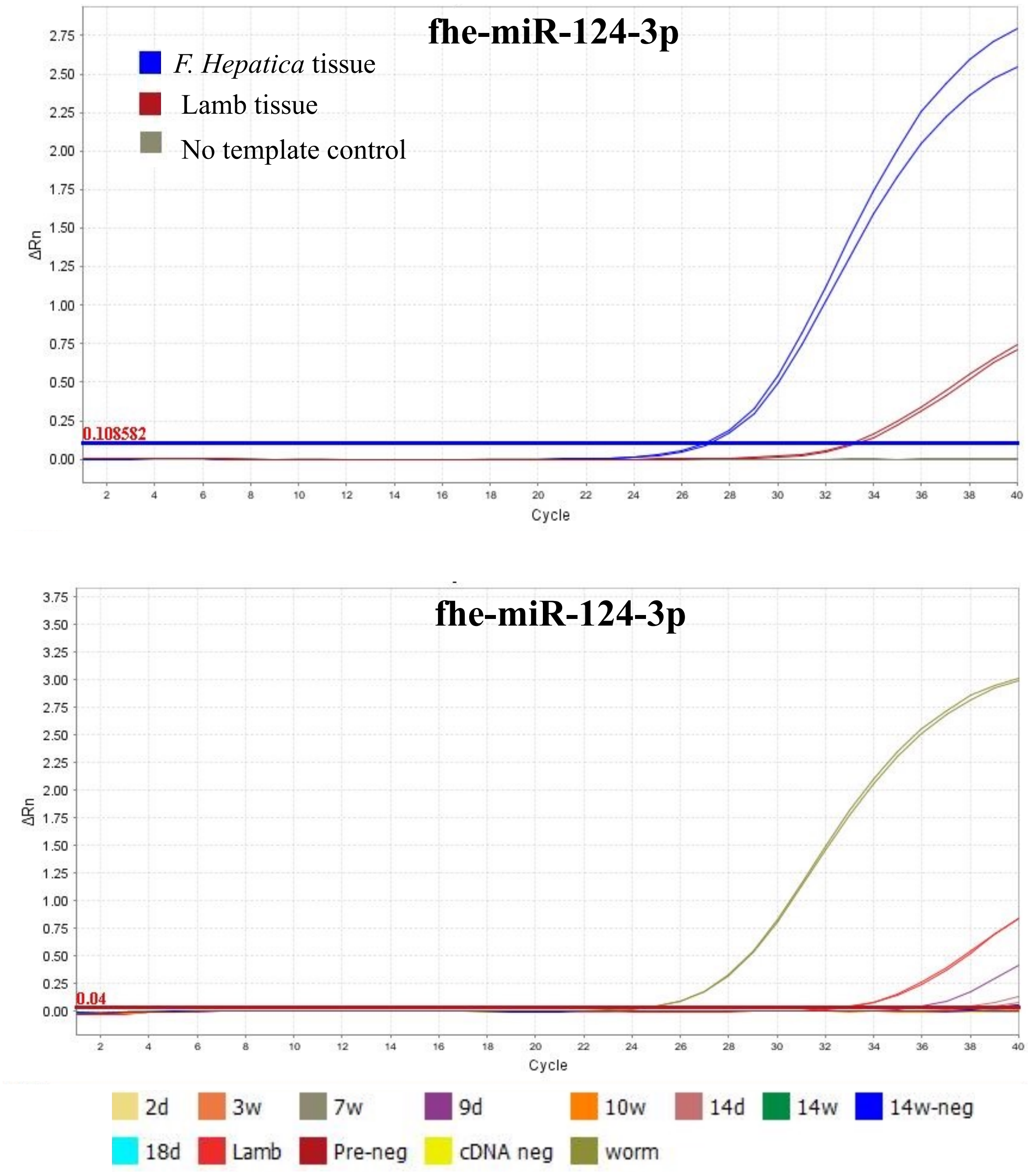


**Supplementary Figure 2. Detection of fhe-miR-124-3p.** RT-qPCR amplification plot showing (A) detection of canonical fhe-miR-124-3p in *F. hepatica* tissue and lamb tissue, (B) canonical fhe-miR-124-3p was hardly amplified or in Set A and B sheep sera samples (n = 6 sheep sera samples pooled at each time point). d: days; w: weeks; Pre-neg: Pre-infection timepoint at day 0; 14w-neg: non-infected age-matched control at 14 weeks. Results of two technical replicates for each sample are shown in the amplification plots.

A

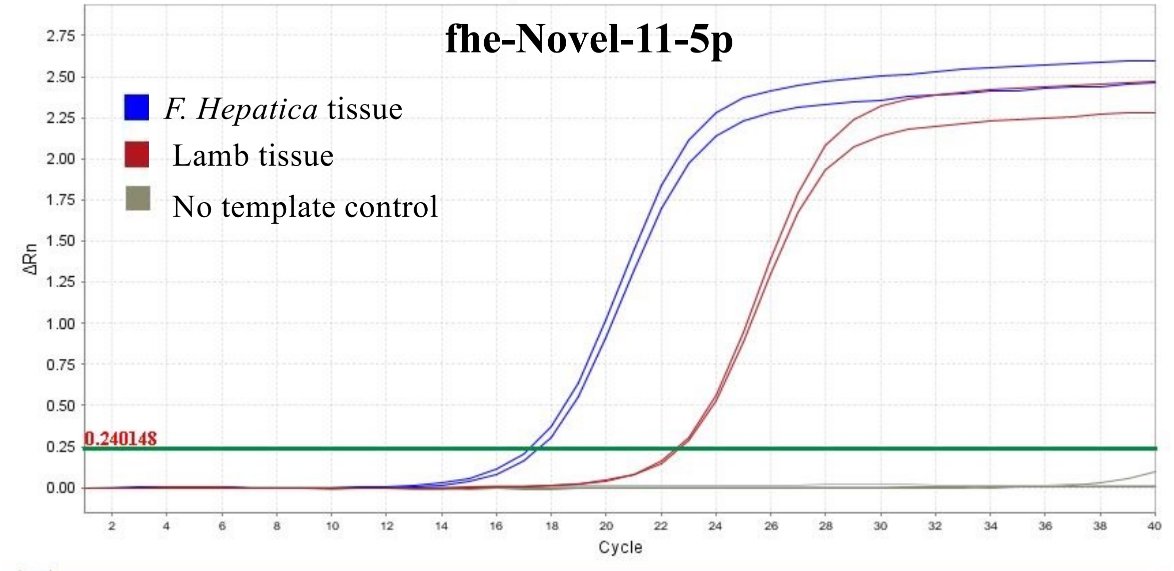


B

**Ovis aries 18S ribosomal RNA gene, complete sequence**

Sequence ID: KY129860.1 Length: 1869 Number of Matches: 1

Range 1: 671 to 688 GenBank Graphics ▼ Next Match ▲

| Score | Expect | Identities | Gaps | Strand |
|---|---|---|---|---|
| 36.2 bits(18) | 0.024 | 18/18(100%) | 0/18(0%) | Plus/Plus |

```
Query  1    AAGCTCGTAGTTGGATCT  18
            ||||||||||||||||||
Sbjct  671  AAGCTCGTAGTTGGATCT  688
```

**Supplementary Figure 3. Detection of fhe-Novel-11-5p** (A) RT-qPCR amplification plot showing amplification of canonical fhe-Novel-11-5p in *F. hepatica* tissue and lamb tissue. Results of two technical replicates for each sample are shown. (B) BLASTN output showing that the shorter Novel-11-5p isomiR sequence found in sheep sera matched 100% with the ribosomal RNA in sheep transcriptome.

**A**

fhe-miR-124-3p   TTAAGGCACGCGGTGAATGTCA
bta-miR-124-3p   TTAAGGCACGCGGTGAATGCCAAG

**B** **PREDICTED: Ovis aries uncharacterized LOC105608520 (LOC105608520), transcript variant X1, ncRNA**

Sequence ID: XR_003588146.2 Length: 819 Number of Matches: 1

Range 1: 658 to 742 GenBank Graphics ▼ Next Match ▲ Previous Match

| Score | Expect | Identities | Gaps | Strand |
|---|---|---|---|---|
| 158 bits(85) | 4e-38 | 85/85(100%) | 0/85(0%) | Plus/Plus |

```
Query  1    AGGCCTCTCTCTCCGTGTTCACAGCGGACCTTGATTTAAATGTCCATACAATTAAGGCAC  60
            ||||||||||||||||||||||||||||||||||||||||||||||||||||||||||||
Sbjct  658  AGGCCTCTCTCTCCGTGTTCACAGCGGACCTTGATTTAAATGTCCATACAATTAAGGCAC  717

Query  61   GCGGTGAATGCCAAGAATGGGGCTG  85
            |||||||||||||||||||||||||
Sbjct  718  GCGGTGAATGCCAAGAATGGGGCTG  742
```

**Supplementary Figure 4. Discovery of the presence of oar-miR-124-3p.** (**A**) Comparison of the miRBase annotated canonical sequences of miR-124-3p of *F. hepatica* (fhe) and *Bos Tauras* (cattle; bta). Red represents nucleotide(s) that did not match between the two species, blue indicates nucleotide(s) missing from the annotated canonical sequence but present in the precursor template of the miRNA. (B) Output from Blastn showing that the precursor of bta-miR-124-3p aligned 100% with the noncoding RNA region of sheep transcript.
